# Mental Imagery for Musical Changes in Loudness

**DOI:** 10.3389/fpsyg.2012.00525

**Published:** 2012-12-03

**Authors:** Freya Bailes, Laura Bishop, Catherine J. Stevens, Roger T. Dean

**Affiliations:** ^1^MARCS Institute, University of Western SydneySydney, NSW, Australia

**Keywords:** mental imagery, loudness, music, motor processing, melody, working memory

## Abstract

Musicians imagine music during mental rehearsal, when reading from a score, and while composing. An important characteristic of music is its temporality. Among the parameters that vary through time is sound intensity, perceived as patterns of loudness. Studies of mental imagery for melodies (i.e., pitch and rhythm) show interference from concurrent musical pitch and verbal tasks, but how we represent musical changes in loudness is unclear. Theories suggest that our perceptions of loudness change relate to our perceptions of force or effort, implying a motor representation. An experiment was conducted to investigate the modalities that contribute to imagery for loudness change. Musicians performed a within-subjects loudness change recall task, comprising 48 trials. First, participants heard a musical scale played with varying patterns of loudness, which they were asked to remember. There followed an empty interval of 8 s (nil distractor control), or the presentation of a series of four sine tones, or four visual letters or three conductor gestures, also to be remembered. Participants then saw an unfolding score of the notes of the scale, during which they were to imagine the corresponding scale in their mind while adjusting a slider to indicate the imagined changes in loudness. Finally, participants performed a recognition task of the tone, letter, or gesture sequence. Based on the motor hypothesis, we predicted that observing and remembering conductor gestures would impair loudness change scale recall, while observing and remembering tone or letter string stimuli would not. Results support this prediction, with loudness change recalled less accurately in the gestures condition than in the control condition. An effect of musical training suggests that auditory and motor imagery ability may be closely related to domain expertise.

## Introduction

Musicians imagine music during mental rehearsal (Holmes, 2005), when reading from a score (Brodsky et al., 2003), and while composing (Covington, 2005; Bailes, 2009; Bailes and Bishop, 2012). An important characteristic of music is its temporality, and among the parameters that vary through time is sound intensity, perceived as patterns of loudness. Desired increases in loudness can be notated in a score as “crescendi,” while decreases can be notated as “decrescendi.” There is anecdotal evidence that imagined music can vary in its overall “loudness” level (Trusheim, 1991; Sacks, 2007), but empirical evidence of imagery for loudness is inconclusive (Intons-Peterson, 1992; Pitt and Crowder, 1992; Wu et al., 2010). Moreover, imagery for changes in loudness remains a relatively neglected topic.

Mental representations of pitch and melody have been shown to involve auditory (Deutsch, 1970; Keller et al., 1995), verbal (Keller et al., 1995), and motor processing (Mikumo, 1994; Finney and Palmer, 2003). Yet, how we represent musical changes in loudness is unclear. In the current study, the modalities that contribute to imagery for loudness change were investigated. Theories suggest that our perceptions of loudness change relate to our perceptions of force or effort, implying that a motor representation is involved. For example, we have proposed a causal chain whereby the literal Force (F) with which a player activates an instrument is transmitted as the physical Energy (E) of the sound. For both player and listener, an appreciation of the Effort (E) involved in this contributes to the perception of Loudness (L) and Arousal (A; Dean and Bailes, 2008, 2010). This proposal (FEELA) was based on computational analyses of patterns of acoustic intensity, and it closely relates to ideas of Todd (1992). Some empirical evidence to corroborate this theory is provided by Eitan and Granot (2006). In an experiment in which participants were asked to imagine a figure moving to musical stimuli, an association was found between crescendi (increase in sound intensity) and the energy of the movements of the imagined figure. For example, figures were imagined moving from a walk to a run as the loudness increased. Following from this theoretical and empirical work, we hypothesize that motor representations contribute to imagery for musical changes in loudness.

The role of motor representations in auditory imagery is critical to distinctions that have been made between an “inner ear” (acoustic imagery) and an “inner voice” (subvocal rehearsal) (Smith et al., 1992). Both have been associated with the working memory subsystem known as the “phonological loop,” involved in processing and memorizing verbal material (Kalakoski, 2001). Articulatory suppression has a negative impact on performance in tasks likely to elicit auditory mental rehearsal, suggesting that subvocalization can contribute to the generation of auditory imagery (Smith et al., 1995). Brodsky et al. (2003) used a musical task that is commonly performed by musicians, namely reading musical notation in silence, to examine the impact of concurrently performing auditory versus phonatory interference tasks on the successful imagining of a notated melody. Phonatory interference by way of concurrently singing or humming a different melody to that in the score proved the most distracting, pointing to acoustic imagery, and subvocal rehearsal in imagery for melodies. However, this research is limited in its focus on imagery for sequences of discrete events (notes), rather than on the dynamic properties of music. It is also shaped by the original concern of working memory models to describe verbal processing, with motor considerations in auditory imagery restricted to vocal production (see also Hubbard, 2010).

In the current study, an experiment was conducted to investigate the modalities that contribute to imagery for loudness change. Investigations of visual imagery and working memory have used an interference paradigm as the means to disrupt different types of processing. For example, in a study of movement imagery in rock climbing, Smyth and Waller (1998) trained participants on two routes, one vertical and the other horizontal. After training, participants imagined climbing the routes under control conditions and with one of three secondary tasks – dynamic visual noise, spatial tapping, or kinesthetic suppression. The secondary or interference tasks affected differentially the duration of horizontal and vertical routes leading the authors to conclude that there are multiple and complex forms of processing action and imaging movement. An investigation of memory span for ballet movements by professional dancers showed no effect of dynamic visual noise as a form of visual suppression on span and, by contrast, a significant effect of a motor interference task on span (Rossi-Arnaud et al., 2004). Pearson et al. (2008) manipulated background luminance during an imagery or a feature-based attention task. Differential effects of background luminance on the two tasks were used to distinguish effects attributable to imagery from those attributable to task instructions (Experiment 4). Following the tradition of an interference paradigm to probe working memory processes, we devised an interference paradigm in which a trial comprises two interleaved memory tasks, designed to test the interference of remembering material from one on the other. Rehearsal is generally required for maintenance of material in short-term memory (Berz, 1995), and the rationale of the current experiment is that such rehearsal will be variously disturbed by material of different modalities. Distractor tasks were designed to differentially place loads on verbal, auditory, and visuo-motor processing. The design requires the concurrent rehearsal of unfamiliar musical (melodies and loudness change scales) and distractor stimuli in working memory.

One of the challenges presented by ubiquitous real-world stimuli such as music is that it is time-varying. Prior studies of mental imagery have investigated more static material such as pictures, objects, or alphanumeric characters. Thus there is a need to evaluate contemporary accounts of imagery in the context of sequential and temporally structured and varied material. In turn, this requires the development of new methods of: stimulus presentation, on-line generative responding, and analysis of the resulting production (time-series) data. In short, investigation of imagery in music demands a method of responding that captures its temporal unfolding, and this may be best achieved by way of a production (rather than recognition) task. Accordingly, we used a continuous response paradigm that encouraged participants to imagine loudness change stimuli. Participants moved a volume slider to indicate increases and decreases in the “loudness” of the imagined stimuli. The advantages of such an approach are twofold. First, enacting the response in time is more likely to recruit a mental image of the stimulus than performing a stimulus recognition task. Second, movement is integral to this response mode, respecting our hypothesized link between representations of intensity and motor effort.

The principal hypothesis was that imagining changes in loudness would be disrupted by concurrently remembering movement sequences (presented visually). However, the loudness change stimuli in the current experiment comprised loudness changes in the sounding of ascending and descending scales, and pitch (the patterns of note ascent and descent) is integral to a representation of such stimuli, so it was possible that tone sequences would also impair mental imagery. Finally, if participants chose a strategy of labeling increases and decreases of intensity as “up” and “down” respectively in order to remember the loudness change scale stimuli, then a concurrent verbal task of remembering letters could be expected to interfere with the task of recreating the loudness change stimuli, perhaps suggestive of a verbal representation rather than a mental image. In line with previous research (e.g., Williamson et al., 2010), we describe letters as verbal stimuli due to their possible encoding in word form.

In experiments on working memory for actions that use an interference paradigm (e.g., Smyth and Pendleton, 1989) there is a problem, rarely discussed, of the similarity of intervening material with to-be-remembered material. For example, greater interference for recalling configurational movements of the body has been observed when intervening material consists of configurational movements of the body than spatial locations. The conclusion is then drawn that configurational movements are coded in working memory by a spatial plus kinesthetic system. However, there is also much greater similarity between the intervening and the to-be-remembered material in the configurational interference condition than in the spatial interference condition. This problem of similarity between to-be-remembered and interference material is addressed in the present experiment by having distractor stimuli that are all dissimilar from the to-be-remembered loudness change scale material.

Conductor gestures were used as visuo-spatial distractor stimuli of relevance to the communication and understanding of musical intensity, and to represent a motor sequence. Action-observation theories would suggest that observing a sequence of conductor gestures necessarily activates motor representations. Simulation theory (see Berthoz, 1996; Grush, 2004) also argues that we observe and understand the actions of others by covertly simulating them. Accordingly, observing a visual sequence of movements with a view to recalling them would involve simulating their production.

It also was important in the current study to separately determine whether these auditory, verbal, and motor distractor tasks would impact on imagery for *melodic* material, as suggested by past research. We hypothesized that imagining melodies would be disrupted by concurrently remembering tone sequences (presented aurally). However, we expected that imagining melody would involve motor processes too, such that having to remember a sequence of movements while performing a test of imagery for melody would also interfere. As when imagining changes in loudness, remembering visually presented letters while attempting to imagine melody could interfere if the letters were encoded sonically rather than visually.

## Materials and Methods

A within-subjects design comprised two different imagery tasks (melodies, loudness change scales), each with four different distractor conditions (control, letter sequence, tone sequence, movement sequence), generating eight different experiment conditions.

### Participants

Participants (*N* = 32, 17 female, 15 male) able to read musical notation were recruited from universities and community music societies in greater Sydney. They received a small travel reimbursement (15 AU$). Ages ranged from 22 to 71 years (*M* = 41.6, SD = 16). Participants had a mean Ollen Musical Sophistication Index (OMSI; Ollen, 2006) of 595 (range 119–993, where a score >500 classifies the participant as “more musically sophisticated” and a score <500 as “less musically sophisticated”), with a mean of 8.7 years of musical training (range 1–16).

### Stimuli

#### Melodies

For the melody imagery task, 28 melodies were selected from the Australian Music Examinations Board (AMEB) aural test syllabus for grades 2–3 (AMEB, 2002). In the current experiment, these melodies were designed to be retained in memory and to be related to a visual score. The melodies were monophonic, between eight and 12 notes in length, and written in a variety of different major and minor keys. All melodies ended on the tonic of the key. The audio files were generated and recorded through a Yamaha Disklavier 3 MIDI (Musical Instrument Digital Interfance) grand piano, controlled by Max/MSP. The velocity of each note was held constant, and each melody was made to span 8 s. Visual scores of the melodies were written in Sibelius.

Half of the melodies were altered to produce the “different” test stimuli, while half were unaltered for the “same” test stimuli. Three types of alteration were made: (1) the order of two consecutive pitches was reversed, as in the “Exchange” comparison of Mikumo (1994; four melodies), (2) a “step” was exchanged for a “leap” (four melodies), or (3) a “leap” was exchanged for a “step” (four melodies). As in Dowling (1978), a step was defined as an interval of three semitones or fewer, and a leap was an interval of four semitones or greater. Within the constraints of each alteration type, changes were designed to be visually non-obvious (e.g., no new accidentals or repeated notes) and avoid introducing dissonance into the melodic context. Contour was disrupted for five of the melodies. Alterations occurred evenly across beginning, middle, and end locations of the melody, but never occurred on the first or last notes.

#### Loudness change scales

For the loudness change imagery task, 16 different loudness change patterns comprising sequences of crescendi and decrescendi were produced. Eight loudness patterns were superimposed on an ascending/descending (in pitch) one octave major scale, while eight were superimposed on a descending/ascending (in pitch) scale. The audio files were generated and recorded through the Disklavier, controlled by Max/MSP. Each note in the scale was 500 ms, so that all the scales spanned 8 s. Half of each scale type (e.g., ascending/descending) began with a crescendo, while the other half began with a decrescendo. Each stimulus comprised between two and four loudness changes (crescendo or decrescendo), lasting between three and eight notes each. Loudness changes were implemented by manipulating the MIDI signal sent from Max/MSP to the Disklavier for the velocity at which each note should be played. The minimum and maximum note velocities were the same for all loudness changes (MIDI note velocity range from 20 to 60). No more than two consecutive notes shared the same note velocity.

For use in the test phase, visual scores of the scales, without loudness change markings, were written in Sibelius. Powerpoint and the screen capture software Capture Me were then used to record videos of each scale being gradually revealed at the rate of one note per 500 ms.

#### Distractor stimuli

##### Letter sequence

The same total set of six letters as used by Williamson et al. (2010) was used to construct four-letter visual sequences. Three letters from this set rhyme (B, D, G) and were expected to be easily confused in phonological memory, while three do not (M, Q, R). “Different” trials at test replaced a letter from the presentation sequence with either a rhyming letter (half) or a non-rhyming letter (half). Letter sequences were created in Powerpoint and recorded as videos using Capture Me. Each letter remained on the screen for 2 s with no gap in-between.

##### Tone sequence

Four-tone sequences were generated in Audacity. Pure sine tones were used, each being 2 s long, presented sequentially with no gap in-between. For each trial, the four tones were selected from outside the key^1^ of the corresponding melody or loudness change scale stimulus. A set of possible tone sequences was constructed for each key prior to the experiment, from which one tone sequence was randomly selected once a melody or scale from the corresponding key was presented. “Different” trials at test replaced a tone from the presentation sequence with a tone also from outside the key of the melody or scale stimulus.

##### Movement sequence

A set of 10 clips of musical conducting were selected from the videos provided in “Expressive Conducting” (Wiens, 2002). The clips were selected to represent varied conducting gestures that ranged from slow to fast, and from small to large. No attempt was made to control the relationship between gestures and the pitch or loudness content of melodic and loudness change scale stimuli. While different gestures might be associated with the communication of different levels of musical sound intensity, the subjective nature of this was beyond the scope of the present study, and so beyond choosing varied gestures, no attempt was made to control for level of expressed intensity in their selection. The movements were recorded from the back right of the conductor such that the face was not visible, and the white baton could be seen against the black background. The baton was visible at all times, and the left hand could not be seen. Original clips that were shorter than 2 s (the shortest original clip was 1.8 s) were stretched in Adobe Premiere Pro CS4 to bring them to the requisite length. Movement sequences were constructed in iMovie. They comprised three silent clips with a 1-s blank (black) screen in-between each clip. For half of the sequences, one of the three clips was replaced by another clip to create a corresponding “different” sequence. While letter and tone distractor sequences comprised four distinct events, pilot testing of gesture sequences suggested no difference in recognition accuracy between three and four gestures. However, in the pilot, participants appeared to be discouraged by the difficulty of remembering a longer sequence. This is comparable to observations from research in working memory for dance movements. Experiments on working memory span for body actions typically report a mean span of three actions for adult participants (e.g., Wood, 2007; Wachowicz et al., 2011).

### Apparatus

**Figure 1:**
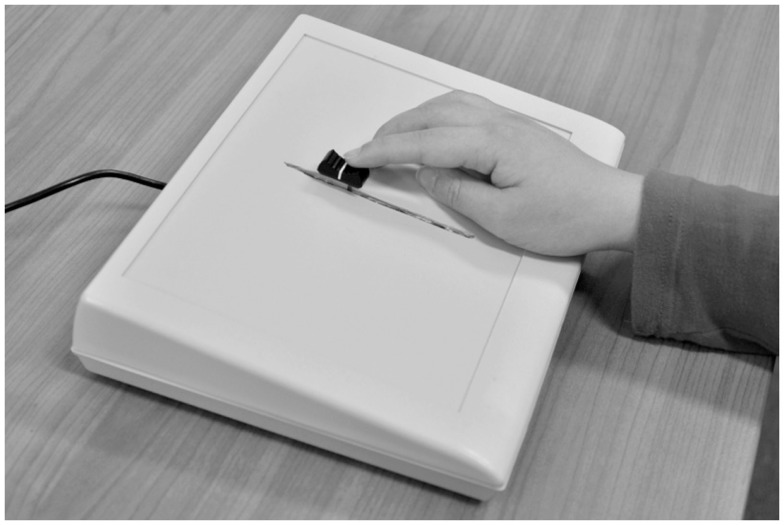
**Slider used to record imagery for loudness change**. Photograph of an I-CubeX push v1.1 slider, used to measure the changes in loudness imagined by participants during the “Imagery for loudness change” task.

The experiment was run from a MacBook (OS X 10.5.8). Stimuli were presented and data were collected using a custom-made patch in Max/MSP. Participants wore Sennheiser HD 650 headphones, and data in the loudness change imagery task were collected by means of an I-CubeX push v1.1 slider facing away from the participant at a slight upwards incline (Figure 1).

### Procedure

The study was approved by the Human Research Ethics Committee of the University of Western Sydney. Written informed consent was first sought to participate in the study, and general instructions about the format of the experiment were provided. Participants began by filling out the OMSI questionnaire. Trials for each of the eight experiment conditions (two imagery tasks × four distractor tasks) were blocked, and instructions specific to the condition were provided at the start of that block. Participants then performed a practice trial for the block, and were given an opportunity to ask the experimenter any questions that they had before proceeding to the experiment trials. Presentation of the eight blocks was random. Each of the 24 melody stimuli was presented once without repeat across melody imagery blocks. Each of the 14 loudness change scale stimuli was presented once or twice across loudness change imagery blocks (the Max/MSP program randomly selected without replacement all 14 stimuli, then began the process again until 10 of the list had been presented a second time).

#### Imagery for melodies

**Figure 2:**
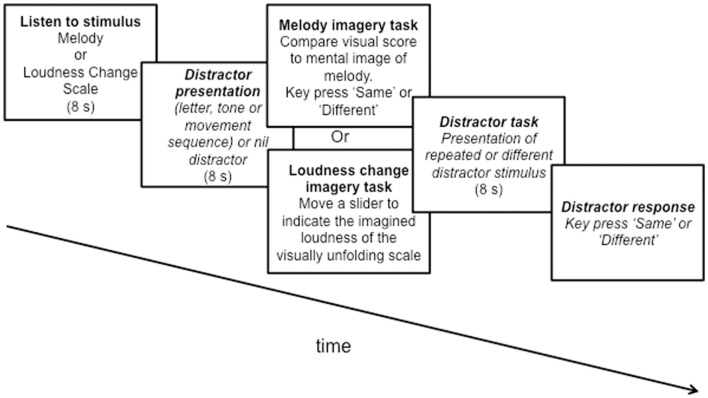
**Details of experimental procedure**. Schematic of an experimental trial. In the imagery for melodies task, the procedure is represented in the upper panel of frame 3. In the imagery for loudness change task, the procedure is represented in the lower panel of frame 3.

In a melody trial, a melody was sounded, followed by presentation of the distractor stimulus (letter sequence, tone sequence, movement sequence, or control period of 8 s). Immediately after the distractor stimulus presentation, a visual score of the melody appeared on screen, and participants indicated as quickly as possible whether the score was the same or different to the melody that they had heard, by comparing their mental image of the melody with the score. “Same” and “Different” buttons appeared next to each other on the screen, and participants used a mouse to indicate their response. Following the melody test, the trial ended for the control condition, or a distractor recognition test appeared, in which participants were presented with the distractor stimulus (letter, tone, or movement sequence) and used the same buttons to indicate whether the distractor test sequence was the same or different to the distractor stimulus which had originally been presented. Figure 2 shows the procedure.

#### Imagery for loudness change

In a loudness change scale trial, a scale modulated in acoustic intensity (loudness change) was sounded, followed by presentation of the distractor stimulus (letter sequence, tone sequence, movement sequence, or control period of 8 s). Immediately after the distractor stimulus presentation, an unfolding visual score of the notes of the scale was presented on the screen, and participants used a volume slider to indicate their mental image of the loudness change profile of the scale that they had heard. Notes appeared on the score at the same pace as they had been sounded at the start of the trial (i.e., one note per 500 ms), and participants were instructed to match the timing of their slider adjustments to the timing of the unfolding visual score. To ensure that slider movements began from the appropriate imagined loudness level at the start of the scale, a 2-s long orientation period was provided, visually marked by a yellow circle on the screen. During this time participants were to move the slider to the level that they thought best represented the opening loudness of the scale, before going on to indicate the loudness changes corresponding to the visually unfolding scale^2^. Following the loudness change scale test, the trial ended for the control condition, or a distractor recognition test appeared, in which participants were presented with the distractor stimulus (letter, tone, or movement sequence) and used “Same” and “Different” buttons to indicate whether the distractor test sequence was the same or different to the distractor stimulus which had originally been presented. Figure 2 shows the procedure.

Each block comprised six trials and a practice. The experiment lasted approximately 45 min.

### Analysis

#### Loudness change scale recall scores

**Figure 3:**
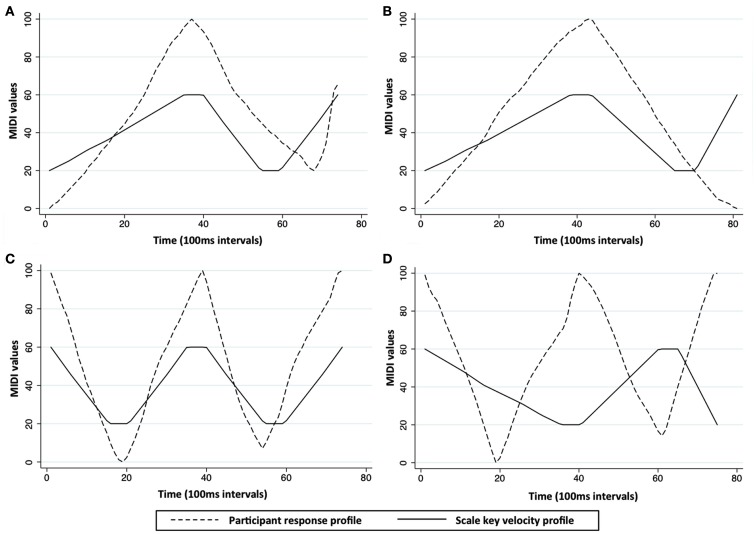
**Sample participant response profiles and scale key velocity profiles**. Each plot shows one participant’s response profile for a single trial and the corresponding scale key velocity profile that the participant was attempting to recall and map out while imagining the scale. DTW provides a measure of the average distance between participant response and scale key velocity profiles per 100 ms time interval. Plots **(A)** and **(C)** show trials in which loudness change profiles were imagined correctly and with precise timing; plots **(B)** and **(D)** show trials in which loudness change profiles were not imagined correctly.

Participant responses for the loudness change scale recall task comprised the series of slider values produced by each participant on each trial. Figure 3 illustrates sample participant response profiles and the corresponding reference scale key velocity profiles for four trials. Each participant’s performance on the task was assessed by measuring the similarity between their response profiles and the corresponding scale key velocity profiles. Dynamic time warping (DTW; Giorgino, 2009) was used to compare participant response profiles and scale key velocity profiles. DTW is suitable for use with time-series data as it does not require independence of data points within the series. It identifies points along test (i.e., participant response) and reference (i.e., scale key velocity) data series that most likely correspond with each other. An average distance between profiles per event is then calculated that is independent of profile length. Participant profiles varied in length, since slider position was sampled continuously (every 100 ms) only when the slider was in motion. Scale key velocity profiles, originally 16 events in length, were therefore stretched so that they were continuous and had the same number of events as each individual participant profile. Temporal relationships between loudness changes were maintained through this step of the analysis. The DTW distance between each participant response profile and corresponding key velocity profile was then calculated. A total of 0.3% of the data from four trials (belonging to three participants) were excluded as outliers as DTW values were greater than 2.5 × SD from the participant’s mean.

#### Melody recognition scores

Accuracy in the melody recognition was calculated as the proportion of correct responses (i.e., correct identification of a different or same stimulus) from all given responses per condition. Four participants were at chance performance only in the nil distractor (control) condition, and consequently they were excluded from analyses on the melody task.

#### Multi-level linear modeling

Multi-level linear modeling was used (lme4 in the statistical program “R”) to determine how well the distractor condition was able to model the scores. One advantage of this approach over ANOVA is the possibility of modeling random effects so that different intercepts and gradients for individuals and block order can be included, thus controlling for intersubject variability or order effects. Models were developed stepwise, using interference condition as a predictor, and testing the impact of individuals, block order, OMSI, and years of musical training as random effects. Model selection used the Bayesian Information Criterion (BIC) to determine the most parsimonious fit. Confidence intervals (CI) were calculated as Highest Posterior Density estimates obtained by Markov Chain Monte Carlo sampling.

## Results

### Imagery for loudness change

**Figure 4:**
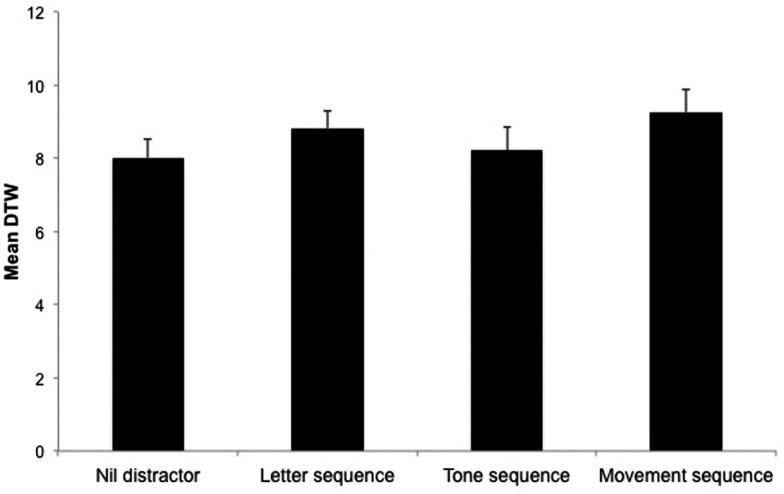
**Mean loudness change scale recall score (DTW)**. Mean DTW distances between slider response and stimulus intensity profiles under different distractor conditions. Small values resemble close loudness change reproduction, i.e., better task performance. Error bars represent standard error.

In the best fit multi-level linear model, recall of loudness change after the movement sequence distractor was significantly worse than recall under the nil distractor condition (β = 1.04, *t* = 2.07, 95% CI: 0.04 to 2.07, *p* = 0.04). Figure 4 displays the DTW distances in each of the distractor conditions. Neither recalling loudness change scales under letter sequence distraction (β = 0.90, *t* = 1.86, 95% CI: −0.07 to 1.84, *p* = 0.06), nor tone sequence distraction (β = 0.42, *t* = 0.87, 95% CI: −0.50 to 1.44, *p* = 0.38) was significantly worse than in the nil distractor control.

The optimized model also included a random intercept for each individual participant (SD = 2.39), for years of musical training (SD = 0.89), and for block order (SD = 0.67).

### Imagery for melodies

**Figure 5:**
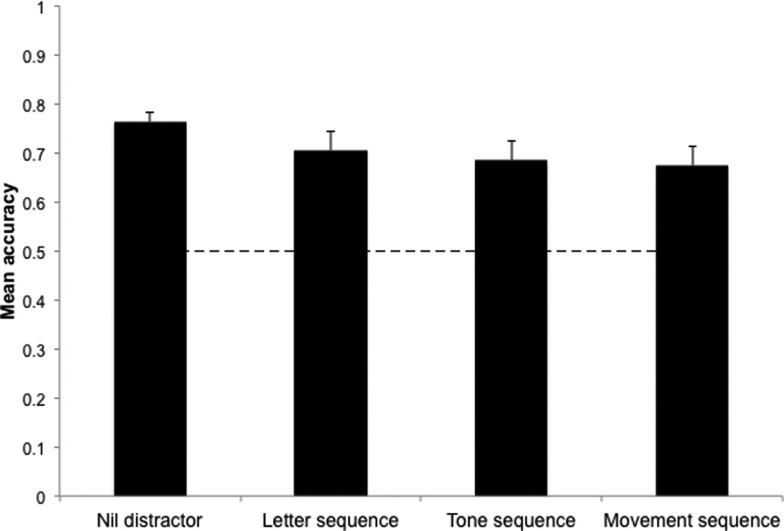
**Mean accuracy in melody recognition under different distractor conditions**. Error bars represent standard error. Dashed line represents chance level accuracy.

In the model of accuracy in the melody recognition task, performance was not significantly different from the nil distractor condition under tone sequence distraction (β = 0.08, *t* = 1.70, 95% CI: −0.17 to 0.03, *p* = 0.09). Neither melody recognition under letter sequence distraction (β = 0.06, *t* = 1.26, 95% CI: −0.16 to 0.04, *p* = 0.21) nor movement sequence distraction (β = 0.09, *t* = 1.91, 95% CI: −0.19 to 0.01, *p* = 0.06) was significantly worse than in the nil distractor control. Results are displayed in Figure 5.

As in the model of DTW distances as an index of imagery for loudness change, the optimized model of accuracy in recognizing melodies included a random intercept for each individual participant (SD = 0.10). Here no significant contribution of years of musical training or block order was found.

Melody recognition in the nil distractor/control condition was significantly better than chance [*t*(26) = 33.5, *p* < 0.0001], making it unlikely that performance in this task was at floor.

### Memory for distractor stimuli

It was also of interest to compare memory for the distractor stimuli following performance in each of the quite different loudness change scale recall and melody recognition tasks. Table 1 summarizes the proportions of correctly remembered letter, tone, and movement sequences.

**Table 1:** **Mean and SD (in parentheses) of accuracy in distractor stimulus recognition**.

	Letter	Tone	Movement
Loudness change scale recall	0.87 (0.1)	0.76 (0.2)	0.55 (0.2)
Melody recognition	0.86 (0.1)	0.74 (0.2)	0.68 (0.2)

During the loudness change scale task, correct recall of the distractor sequences differed significantly by distractor type, as assessed by a repeated measures ANOVA of accuracy in the distractor task, *F*(2,94) = 28.11, *p* < 0.001. Planned contrasts using a Bonferroni adjusted α of 0.02 show that letter sequences were recognized better than tone sequences *t*(31) = 2.64, *p* < 0.01, which were recognized better than movement sequences, *t*(30) = 6.94, *p* < 0.001.

The correct recall of distractor sequences in the melody recognition task also differed significantly by distractor type, *F*(2,94) = 8.11, *p* < 0.001. Once again, letter sequences were recognized better than tone, *t*(31) = 3.16, *p* < 0.01, and movement sequences *t*(30) = 4.56, *p* < 0.001, but accuracy recognizing tone and movement sequences did not differ, *t*(30) = 1.16, *p* = 0.05.

### Imagery and musical experience

No relationship was found between OMSI and score when modeling performance in either the melody recall or loudness change scale reproduction tasks. The OMSI is designed to categorize participants as more (>500) or less (<500) musically sophisticated. Comparing the imagery scores of participants categorized in this way confirmed the result from linear modeling that there were no significant differences on either imagery task along this dimension. However, years of musical training contributed to the model of accuracy in loudness change scale reproduction. Furthermore, a positive correlation of years of musical training with accuracy on the melody imagery task was found *r*(30) = 0.4, *p* < 0.05 (two-tailed).

## Discussion

This experiment aimed to determine the disruptive effects of rehearsing letter, tone, and movement sequences on mental imagery for changes in loudness. As predicted, rehearsing a movement sequence in mind significantly impaired the recall of loudness change scales. Rehearsing tone sequences did not, though rehearsing letter sequences, a task which could have involved subvocal motor rehearsal, came close to producing a significant impairment. Analyses of how well participants were able to remember the different distractor stimuli revealed that movement sequence recognition was consistently weaker than the recognition of the other distractor sequences. Equating the difficulty of tasks that are to be used in working memory experiments is a vexed issue that receives relatively little discussion. While recognizing conductor gestures might be regarded as more difficult, it is just as likely that the stimuli and task are less familiar than performing a task containing letters or musical tones. Familiarity refers to having knowledge of the material in long-term memory. Thus familiarizing participants with novel material such as gestures within an experiment is one way in the future that could strengthen task comparability. Alternatively, unfamiliar words and tones could be used to be more comparable with the novel conducting gestures.

It seems likely that retaining the movement sequences presented a substantial cognitive load during the performance of any concurrent memory task. However, memory for melodies was only marginally impaired by the movement distractor task, and so its impact primarily concerned the specific task of reproducing imagined changes in loudness. While a motor response was required to reproduce these imagined loudness changes, evidence from a separate experiment suggests that mentally rehearsing the movement sequences does not impair use of the slider *per se*. Consequently, our experiment provides evidence that imagery for musical loudness change can involve motor processing.

Contrary to expectations, the accurate imagining of melodies was not significantly impaired by the concurrent rehearsal of tone sequences. Perhaps participants ignored the intervening tone sequence, choosing to prioritize mental rehearsal of the melodies. Such a strategy would have been associated with poor performance on the subsequent tone recognition task, yet accuracy was better than chance [*t*(31) = 5.32, *p* < 0.001]. In addition, the verbal sequence did not impair melody recognition, a finding which is at odds with the results of Keller et al. (1995). Indeed, no differences in melody recognition were observed between the different distractor conditions.

The retention of letter sequences was significantly higher than the retention of other distractor sequences during both the loudness change scale and melody tasks. Yet this superior letter recognition did not come at the price of memory for the loudness change scale or melodies. The letters were presented visually, but they were selected in the knowledge that they might be encoded phonologically and rehearsed as an acoustic image or by subvocal rehearsal. An absence of interference from the letters in the melody recognition task might suggest a visual rehearsal strategy, while a lack of significant interference in the loudness change scale recall task might point to a similar approach, with the interesting corollary that if letter sequences were rehearsed visually, the successful imagining of loudness change scales must be achieved as a motor or auditory image, and not as a visual image of crescendo and decrescendo markings.

The finding that imagery for musical changes in loudness is disrupted by the concurrent rehearsal of a movement goes some way to answering the question of whether patterns of musical loudness are best described as a verbal, auditory, or motor representation. To be added to the list of potential modalities is vision, given that the presentation of the conductor gestures was visual, and participants might have been translating the changes in loudness that they heard into a visual code of what are called “hair pins” (score annotations to indicate crescendi and decrescendi). The absence of an impairment from rehearsing tones does not seem to suggest an exclusively auditory image. Similarly, the lack of a statistically significant impairment from rehearsing a letter sequence does not point to a uniquely verbal labeling of loudness change information such as “up,” “down.” The most likely scenario is that a balance of representation modality was involved. Such a view is consistent with current accounts of working memory that emphasize interference from process rather than content; these accounts recognize the influence of task demands, task relevant, and task irrelevant information, instructions, and context on performance (e.g., Marsh et al., 2009).

Working memory involves simultaneous short-term storage and processing of information (Oberauer, 2009) and is limited to three to five meaningful items in adults (Cowan, 2010). Individual differences in working memory capacity are thought to relate to differences in maintenance and retrieval. More specifically, working memory limitations arise from differences in both ability to actively maintain information and ability to retrieve task relevant information in the presence of highly interfering or irrelevant information (Unsworth and Engle, 2007). Research also points to considerable variation in imagery abilities (Mast and Kosslyn, 2002; Keller and Koch, 2006), and the current study is consistent with this, as models of performance on both types of imagery task (loudness change and melody) were improved by accounting for the variability introduced by individual participants.

Participants in this study were able to read music, suggesting at least a minimal amount of musical training. Not only has a link been established between auditory imagery abilities and musical training (Aleman et al., 2000), this has been extended to the particular context of action-effect anticipation (Keller and Koch, 2008). This suggests that a tight sensorimotor coupling results from extensively rehearsed associations between an action and its consequent sound. In the current study, the participants were not required to physically produce the test stimuli, and so had not explicitly learned an association between movement and melody or loudness change scale items. Nevertheless, musical experience has been found to enhance action-effect coupling quite broadly (Keller and Koch, 2008), and in the current experiment this might have reinforced the ability of participants to imagine motor and auditory components of loudness change. Indeed, the optimal model of performance in the imagery for loudness change task included years of musical training. A correlation between years of musical training and accuracy in the melody imagery task was also found.

Audio-motor coupling has been argued to be strong for musicians, and Baumann et al. (2007) have suggested that the activation of both auditory and motor areas of the brain while listening or playing even when participants attend to a distractor task is evidence for direct and automatic connections between auditory and motor areas in music. In an interview study of experienced musicians, Holmes (2005) found that motor imagery was a significant part of learning and memorizing music for performance. It is interesting that years of training was a significant factor, which amounts to an index of performance experience, while OMSI score was not, which is a composite measure of musical sophistication, taking into account listening and compositional experience. While the current study relied on musically literate participants, it would be interesting to investigate the modalities involved in imaging musical loudness change for a wider population. Some evidence points to a common metaphorical association of movement with musical loudness change (Eitan and Granot, 2006).

In conclusion, we have presented behavioral evidence for motor processing in the imagining of musical changes in loudness. Although concurrent verbal and auditory distractor tasks did not significantly impair participants’ ability to imagine loudness change stimuli, we should not conclude that a uniquely motor representation drives imagery for musical loudness change. These verbal and auditory distractor tasks failed to impair performance on a melody imagery task, in spite of previous research to suggest that melodic material is rehearsed as an auditory image. Future work is needed to determine how rehearsing the tonal and letter sequences employed in the current study should have impaired auditory and verbal imagery for musical stimuli. Individual differences in imagery ability were evident, and it remains to be seen whether these individual differences are reflected in the processing modalities preferred when imagining musical stimuli. Our ongoing research is studying the use of mental imagery for loudness change by expert musicians in performance. The current experiment has provided an effective interference task for loudness change imagery in the guise of conductor gestures, allowing us to examine the strategies used by musicians when they cannot consciously plan (imagine) their expressive intentions.

## Conflict of Interest Statement

The authors declare that the research was conducted in the absence of any commercial or financial relationships that could be construed as a potential conflict of interest.

## Footnotes

^1^A musical key is described by the scale to which most of the notes in a piece of music conform. For example, if most of the notes in a piece are in the scale of C major, with important pitches such as “C” (tonic) or “G” (dominant) occurring particularly often, its key is probably C major.

^2^Since moving a slider is a motor task, it was important to ensure that any impaired performance associated with movement sequence distractor conditions could not be attributable to physical motor production demands. A separate experiment required 12 participants (six from the current experiment and six new participants) to use the slider to indicate the loudness changes that they were hearing in the moment. Since this perceptual task did not require participants to rehearse or recall loudness change, we did not expect any interference from concurrent distractor conditions. Indeed, the participants’ accuracy in marking loudness changes under letter and movement sequence conditions was not significantly different (*z* = −0.235, *p* = 0.81), suggesting that any relative impairment we might find from rehearsing movement sequences in the current imagery experiment could not be attributable to the motor production of the task.
